# Reduced vs. standard dose native *E. coli*-asparaginase therapy in childhood acute lymphoblastic leukemia: long-term results of the randomized trial Moscow–Berlin 2002

**DOI:** 10.1007/s00432-019-02854-x

**Published:** 2019-03-06

**Authors:** Alexander Karachunskiy, Gesche Tallen, Julia Roumiantseva, Svetlana Lagoiko, Almira Chervova, Arend von Stackelberg, Olga Aleinikova, Oleg Bydanov, Lyudmila Bajdun, Tatiana Nasedkina, Natalia Korepanova, Sergei Kuznetsov, Galina Novichkova, Marina Goroshkova, Dmitry Litvinov, Natalia Myakova, Natalia Ponomareva, Evgeniya Inyushkina, Konstantin Kondratchik, Julia Abugova, Larisa Fechina, Oleg Arakaev, Alexander Karelin, Vladimir Lebedev, Natalia Judina, Gusel Scharapova, Irina Spichak, Anastasia Shamardina, Olga Ryskal, Alexander Shapochnik, Alexander Rumjanzew, Joachim Boos, Günter Henze, for the ALL-MB study group

**Affiliations:** 1 Federal Research Centre of Pediatric Hematology, Oncology and Immunology, Moscow, Russia; 2 Department of Oncology, Hematology and Radiotherapy, Pirogov Russian National Research Medical University, Moscow, Russia; 3 Department of Pediatric Oncology/Hematology, Charité-Universitätsmedizin Berlin, Berlin, Germany; 4 Department of Pediatric Oncology/Hematology, Universitätsklinikum Hamburg-Eppendorf, Hamburg, Germany; 5 Department of Paediatrics, Cumming School of Medicine, Faculty of Medicine, University of Calgary, Calgary, Canada; 6 Republic Clinical Research Centre for Pediatric Oncology and Hematology, Minsk, Belarus; 7 Russian Children Clinical Hospital, Moscow, Russia; 8 National Research University Higher School of Economics, Moscow, Russia; 9 Department of Pediatric Hematology, Municipal Children,s Clinical Hospital, Novokuznetsk, Russia; 10 Moscow Regional Oncological Dispensary, Balashikha, Russia; 11 Department of Pediatric Hematology, Morozov Children’s Hospital, Moscow, Russia; 12 Paediatric Oncohematological Centre, Regional Children’s Hospital, Ekaterinburg, Russia; 13 Department of Pediatric Oncology/Hematology, Regional Children’s Hospital, Krasnodar, Russia; 14 Department of Pediatric Oncology/Hematology, Regional Children’s Hospital, Voronezh, Russia; 15 Department of Pediatric Oncology/Hematology, Regional Children’s Hospital, Nizhnevartovsk, Russia; 16 Paediatric Oncohematological Centre, Regional Children’s Hospital, Chelyabinsk, Russia; 17 Department of Pediatric Hematology, Regional Children’s Hospital, Nizhniy Novgorod, Russia; 18 Department of Pediatric Oncology/Hematology, Regional Children’s Hospital, Perm, Russia; 19 Department of Pediatric Oncology/Hematology, Regional Oncological Hospital, Orenburg, Russia; 20 Department of Pediatric Hematology and Oncology, University Children’s Hospital Muenster, Münster, Germany; 21 Institute of Oncology, Radiology and Nuclear Medicine, Dmitry Rogachev National Research Center of Pediatric Hematology, Oncology and Immunology, 1 Samory Mashela str, 117997 Moscow, Russia

**Keywords:** Acute lymphoblastic leukemia, Children, Native *Escherichia coli*-derived asparaginase, Multicenter trial

## Abstract

**Purpose:**

Favorable outcomes were achieved for children with acute lymphoblastic leukemia (ALL) with the first Russian multicenter trial Moscow–Berlin (ALL-MB) 91. One major component of this regimen included a total of 18 doses of weekly intramuscular (IM) native *Escherichia coli*-derived asparaginase (*E. coli*-ASP) at 10000 U/m^2^ during three consolidation courses. ASP was initially available from Latvia, but had to be purchased from abroad at substantial costs after the collapse of Soviet Union. Therefore, the subsequent trial ALL-MB 2002 aimed at limiting costs to a reasonable extent and also at reducing toxicity by lowering the dose for standard risk (SR−) patients to 5000 U/m^2^ without jeopardizing efficacy.

**Methods:**

Between April 2002 and November 2006, 774 SR patients were registered in 34 centers across Russia and Belarus, 688 of whom were randomized. In arm ASP-5000 (*n* = 334), patients received 5000 U/m^2^ and in arm ASP-10000 (*n* = 354) 10 000 U/m^2^ IM.

**Results:**

Probabilities of disease-free survival, overall survival and cumulative incidence of relapse at 10 years were comparable: 79 ± 2%, 86 ± 2% and 17.4 ± 2.1% (ASP-5000) vs. 75 ± 2% and 82 ± 2%, and 17.9 ± 2.0% (ASP-10000), while death in complete remission was significantly lower in arm ASP-5000 (2.7% vs. 6.5%; *p* = 0.029).

**Conclusion:**

Our findings suggest that weekly 5000 U/m^2^*E. coli-*ASP IM during consolidation therapy are equally effective, more cost-efficient and less toxic than 10000 U/m^2^ for SR patients with childhood ALL.

**Electronic supplementary material:**

The online version of this article (10.1007/s00432-019-02854-x) contains supplementary material, which is available to authorized users.

## Introduction

Since Oettgen et al. have first described the antileukemic effects of L-asparaginase (ASP) in 1967, the bacterial enzyme has become a mainstay in both remission induction and post-induction consolidation (Oettgen et al. 1967). Its use in combination with multiagent chemotherapy resulted in a significant increase of long-term event-free survival (EFS) for children and adolescents with acute lymphoblastic leukemia (ALL) over the last decades.

Dependent on various factors, such as origin (*Escherichia coli*-derived ASP, *E. coli*-ASP or *Erwinia chrysanthemi-*derived ASP), preparation (native *E. coli*- or *Erwinia*-ASP, pegylated *E. coli*-ASP [PEG-ASP] or recombinant ASP), dosing, and route of administration (intramuscular, IM vs. intravenous, IV), ASP is regularly associated with adverse effects. Life-threatening side effects may be thrombosis, necrotizing pancreatitis, and hypersensitivity reactions (HSR) due to antibodies directed against ASP, the latter especially after prolonged and IV administration (Schmiegelow et al. 2016; Ko et al. 2015). HSR represent by far the most frequent cause of treatment discontinuation or delays and product shifts, thereby jeopardizing efficacy of therapy and resulting in additional treatment costs (Ko et al. 2015). Also, ASP-related myelosuppression requiring dose reductions of other antineoplastic agents during consolidation has been reported (Oehlers et al. 1969; Kolarz and Pietschmann 1971; Johnston et al. 1974; Merryman et al. 2012).

Because of its lower immunogenicity and longer half-life, similar antileukemic efficacy and levels of asparagine serum depletion when compared to the native preparations, multiple groups recommend the PEG formulation for frontline treatment (Rizzari et al. 2006, 2013; Rizzari 2015; Pieters et al. 2011; Tong et al. 2014; Place et al. 2015).

Therapy aims at achieving prolonged or even complete depletion of serum asparagine. Nevertheless, despite the long-term use of ASP, optimal route of administration and dosing as well as most appropriate second-line treatment remain matters of debate (Rizzari et al. 2013; Place et al. 2015; Alrazzak et al. 2016; Beaupin et al. 2017). According to Western European protocols for childhood ALL, ASP is mostly given IV, whereas the IM route is preferred in most US protocols. The pharmacology varies considerably between these modalities, in particular regarding the half-life of the enzyme, which is longer after IM administration (Ho et al. 1981; Narta et al. 2007; Boos et al. 1996). ASP has been given IM at variable single doses up to 30,000 U/m^2^. Compared to other drugs used for treatment of childhood ALL, ASP is relatively costly. Expenditures differ between preparations (PEG-ASP is more expensive than native ASP), origin (*Erwinia*-ASP is more expensive than *E. coli*-ASP), and protocol design (total cumulative dose administered). Cost-efficient treatments have come to focus not only in countries with low income.

In 1986, Clavell et al. published favorable results with four-agent induction and intensive ASP (Clavell et al. 1986). In the first Russian multicenter protocol Moscow–Berlin (ALL-MB) 91, we chose a less intensive regimen and used 10,000 U/m^2^*E.-coli*-ASP IM weekly during consolidation. The aim was to achieve high EFS rates with side effects that are manageable in local health care facilities (Karachunskiy et al. 2008). According to the ALL-MB91 risk definition, standard risk (SR−) patients comprised about 70% of the total cohort and achieved high EFS rates (over 70%). For the subsequent trial, MB 2002, encouraged by the positive result of MB 91 and also in the pursuit of an economic therapy, we investigated whether a lower dose of ASP for SR patients would reduce costs and possibly also toxicity without jeopardizing efficacy of the treatment. Hence, SR patients in remission were randomized to receive ASP either at a weekly standard dose of 10,000 U/m^2^ or a reduced dose of 5000 U/m^2^ IM during consolidation.

## Patients and methods

### Study design

The study ALL-MB 2002 was designed as a prospective, multicenter randomized trial addressing several questions. The treatment overview is shown in Supplementary Fig. 1. After diagnosis, all patients were randomized to receive either dexamethasone (DEXA) or methylprednisolone (MePRED) during remission induction (Karachunskiy et al. 2015). For patients achieving complete remission (CR), two additional randomizations were performed. Eligible for the ASP-randomization were only SR patients who had to meet all of the following criteria: White blood cell count (WBC) < 50,000/mm^3^, no T-cell immunology, no central nervous system (CNS) leukemia, age at diagnosis > 1 year, no *t*(4;11) or *t*(9;22), and being in CR on day 36. According to this definition, the SR group comprised 70% of the total cohort enrolled in the trial. Following induction chemotherapy, all eligible patients, irrespective of the glucocorticoid administered during induction, were randomly assigned to one of two ASP-regimens during three consolidation phases. Patients received a total of 18 doses of *E. coli*-ASP (Asparaginase medac™) at 10,000 U/m^2^ (ASP-10000) or 5000 U/m^2^ (ASP-5000) IM at weekly intervals.

### Statistical analyses

The study was planned as a non-inferiority study. To confirm that the outcome in arm ASP-5000 was not inferior to that in ASP-10000 (power 80%, *p* < 0.05), the calculated number of patients required for randomization was 304 per group assuming a non-inferiority limit of < 10%. Estimates of disease-free survival (DFS) and overall survival (OS) probability (± standard error) were calculated from the date of randomization to the date of an event (DFS: relapse, death, secondary malignancy; OS: death of any cause). Data were visualized using the Kaplan–Meier method and compared by log-rank test. Cumulative incidences (CI) were estimated according to Kalbfleisch and Prentice. Plots and statistical comparisons were performed using Gray’s test. Follow-up monitoring was based on semi-annual questionnaires.

Toxicity comparison was based on regular reports on side effects provided by the treatment centers participating in the trial. Analyzed side effects included those requiring either a change to another ASP preparation or discontinuation of ASP therapy, respectively, such as severe HSR (anaphylaxis), pancreatitis and thrombosis.

Frequencies and distributions were compared using the *χ*^2^ or Fisher’s exact test. For calculations, Graphpad Prism version 3.0 (Graphpad Software Inc., San Diego, CA, USA), Statistica version 7.0 (Statsoft Inc., Tulsa, OK, USA) and R version 2.4.0 were used on a database closed May 1, 2016.

### Patients

**Fig. 1:**
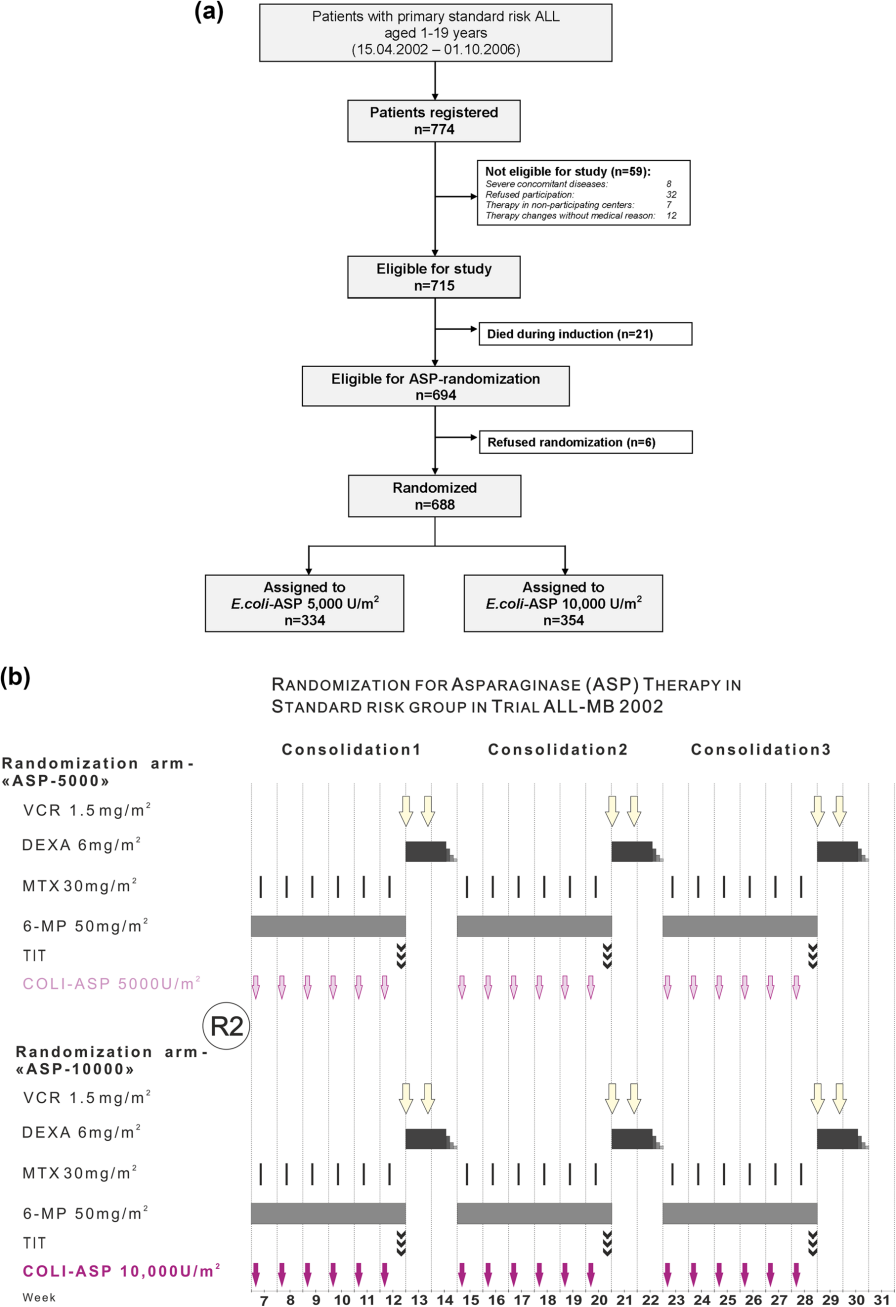
**a** Consort diagram showing recruitment, eligibility, and randomization of standard risk group patients for treatment with 5000 vs. 10,000 U/m^2^ of *E. coli*-asparaginase (*E. coli*-ASP) given intramuscularly during consolidation therapy in trial ALL-Moscow–Berlin 2002 (for details, see main text). **b** Treatment overview: randomization arms ASP-5000 and ASP-10000 in trial ALL-MB 2002

Between April 18, 2002, and October 1, 2006, 774 unselected, consecutive SR patients, aged 1–19 years with newly diagnosed precursor B- or T-cell ALL were registered in 34 centers across Russia and Belarus after approval by local ethics committees. Written informed consents for trial-participation and randomization were obtained from parents and patients, if applicable, or their legal guardians according to the Declaration of Helsinki. The consort diagram is shown in Fig. 1a. Participation was refused by 32 patients, seven patients preferred not to be treated in any of the participating centers. Eight patients were excluded due to severe concomitant diseases, and for 12 patients therapy was changed without medical reason, leaving 715 patients eligible for study. Twenty-one patients died during induction, and six patients refused the randomization. Thus, a total of 688 patients were randomized to either arm ASP-5000 or ASP-10000. All other treatment elements were identical; the overview is shown in Fig. 1b.

### Definitions

Diagnostic criteria and procedures have been described elsewhere (Karachunskiy et al. 2008, 2015). CNS involvement was defined as > 5 nucleated cells/µl cerebrospinal fluid (CSF) with clearly detectable leukemic blasts in the absence of blood contamination, or as leukemic CNS-infiltration disclosed by imaging (CT or MRI), respectively. TEL/AML1, BCR/ABL-, and MLL/AF4 fusion transcripts were identified by cytogenetics or molecular genetics. SR criteria have been described above. CR was diagnosed in the absence of any evidence of leukemia (i.e., normal CSF and a regenerating bone marrow (BM) with < 5% leukemia cells). Relapse was defined as reappearance of leukemia at any site.

### Treatment

At diagnosis, all patients were randomized to receive either DEXA at a dose of 6 mg/m^2^/day orally (PO) or MePRED 60 mg/m^2^/day (PO). Treatment was given as shown in Table 1. After remission was achieved, SR patients were additionally randomized to either arm ASP-5000 or ASP-10000 (18 doses of asparaginase 5000 U/m^2^ or 10,000 U/m^2^ given weekly IM; exclusively Asparaginase medac™ was used). In the case of anaphylaxis, *E. coli*-ASP was discontinued. Patients received three consolidation courses, differing only concerning the asparaginase dose (Fig. 1b; Table 1). After completion of consolidation, maintenance therapy was given for 1.5 years (Table 1).

**Table 1:** Treatment protocol of the ALL-MB 2002 trial for standard risk patients

Phase	Dose and route	Days
Induction		
Dexamethasone^a^	6 mg/m^2^ PO	1–28
Methylprednisolone^a^	60 mg/m^2^ PO	1–28
Vincristine	1.5 mg/m^2^ IV	8, 15, 22, 29, 36
Daunorubicin	45 mg/m^2^ IV	8 (22)^b^
Consolidation I		
6-Mercaptopurine	50 mg/m^2^ PO	43–84
Methotrexate	30 mg/m^2^ IM	43, 50, 57, 64, 71, 78
***E. coli*-asparaginase**^**c**^	**10,000 U**/**m**^**2**^**IM**	**44, 51, 58, 65, 72, 79**
	**5000 U**/**m**^**2**^**IM**	**44, 51, 58, 65, 72, 79**
Dexamethasone	6 mg/m^2^ PO	85–95
Vincristine	1.5 mg/m^2^ IV	85, 92
Consolidation II		
6-Mercaptopurine	50 mg/m^2^ PO	99–140
Methotrexate	30 mg/m^2^ IM	99, 106, 113, 120, 127, 134
***E. coli*-asparaginase**^**c**^	**10,000 U**/**m**^**2**^**IM**	**100, 107, 114, 121, 128, 135**
	**5000 U**/**m**^**2**^**IM**	**100, 107, 114, 121, 128, 135**
Dexamethasone	6 mg/m^2^ PO	141–151
Vincristine	1.5 mg/m^2^ IV	141, 148
Consolidation III		
6-Mercaptopurine	50 mg/m^2^ PO	155–196
Methotrexate	30 mg/m^2^ IM	155, 162, 169, 176, 183, 190
***E. coli*-asparaginase**^**c**^	**10,000 U**/**m**^**2**^**IM**	**156, 163, 170, 177, 184, 191**
	**5000 U**/**m**^**2**^**IM**	**156, 163, 170, 177, 184, 191**
Dexamethasone	6 mg/m^2^ PO	197–207
Vincristine	1.5 mg/m^2^ IV	197, 204
Preventive CNS therapy (age adjusted)^d^		
Methotrexate IT	8/10/12 mg	1, 8, 15, 22, 29, 36, 85, 141, 197, 253, 309, 365, 421
Cytarabine IT	20/26/30 mg	
Prednisone IT	6/8/10 mg	
CNS radiation therapy	– (any age)	
Maintenance therapy		
6-Mercaptopurine (once daily)	50 mg/m^2^ PO	Weeks: 31–36, 39–44, 47–52, 55–60, 63–68, 71–76, 79–84, 87–92, 95–104
Methotrexate (once weekly)	30 mg/m^2^ IM	
Dexamethasone	6 mg/m^2^ PO	Weeks: 37–38, 45–46, 53–54, 61–62, 69–70, 77–78, 85–86, 93–94
Vincristine	1.5 mg/m^2^ IV × 2	

*ALL-MB 2002* the protocol acute lymphoblastic leukemia—Moscow–Berlin 2002, *BM* bone marrow, *CNS* central nervous system, *IM* intramuscular, *ImRG* intermediate risk group, *IT* intrathecal, *IV* intravenous, *PO* per os, *SRG* standard risk group

Bold indicates the differences in treatment between the two compared groups

^a^Glucocorticoid type according to randomization arm

^b^Given on day 22 to SRG patients with ≥ 10% BM blasts on day 15

^c^l-asparaginase dosage according to randomization arm

^d^Age adjusted IT doses: ≥ 1–< 2 years/≥ 2–< 3 years/≥ 3 years

## Results

Of the 688 patients eligible for randomization, 334 were randomized to arm ASP-5000 and 354 to arm ASP-10000 (Fig. 1; Table 2). No significant differences were found between arms regarding gender, age, initial WBC, splenomegaly or genetic aberrations. Patients were also equally distributed with respect to glucocorticoid randomizations and did not differ in early response to therapy (Table 2). Median follow-up of patients remaining in CR was 10.61 years (interquartile range (IQR) 10.08–11.13).

**Table 2:** Patient characteristics by randomization arm

Total patients	Arm ASP-5000	Arm ASP-10000	*р*
	*n* = 334	*n* = 354	
Gender			
Boys	181	192	*0.949*
Girls	153	162	
Age (years)			
≥ 1–< 5	161	189	*0.370*
≥ 5–<10	93	92	
≥ 10	80	73	
Initial WBC count			
< 10,000/µl	210	227	*0.271*
≥ 10,000–< 30,000/µl	102	94	
≥ 30,000–< 50,000/µl	22	33	
Spleen enlargement below left costal margin (cm)			
< 4	258	256	*0.162*
≥ 4	76	98	
Genetics^a^			
*t*(12;21)	29	24	*0.491*
Response to treatment on day 8^b^			
< 1000 leukemic blasts/µl PB	318	331	*0.473*
≥ 1000 leukemic blasts/µl PB	9	14	
Response to treatment on day 15^c^			
< 10% leukemic blasts in BM	272	285	*0.790*
≥ 10–< 25% leukemic blasts in BM	40	39	
≥ 25% leukemic blasts in BM	19	24	
Induction therapy			
Dexamethasone 6 mg/m^2^	168	176	*0.874*
Methylprednisolone 60 mg/m^2^	163	177	

*ASP* asparaginase, *BM* bone marrow, *IM* intramuscular, *PB* peripheral blood, *WBC* white blood cell count

^a^Investigation of relevant translocations performed in 506 patients (*n* = 251 in the arm ASP-5000, *n* = 255 in the arm ASP-10000)

^b^Treatment response on day 8 was documented for 672 patients (327 patients in the arm ASP-5000, 345 patients in the arm ASP-10000)

^c^Treatment response on day 15 was documented for 679 patients (331 patients in the arm ASP-5000, 348 patients in the arm ASP-10000)

**Fig. 2:**
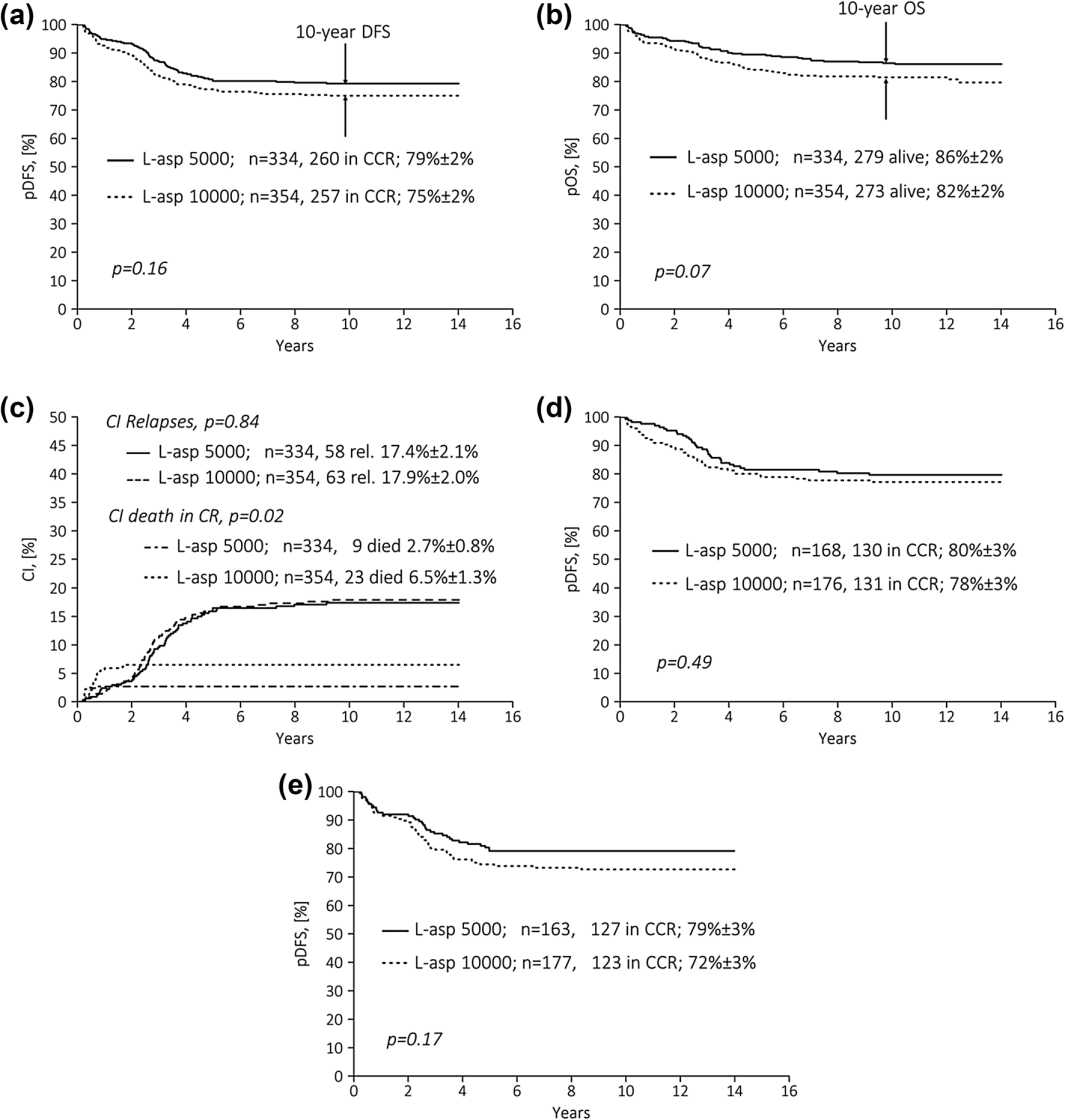
Treatment results (“intent-to-treat-analysis”) for standard risk group patients after consolidation therapy with 5000 vs. 10,000 U/m^2^ of *E. coli*-ASP IM in trial ALL-Moscow–Berlin 2002 by randomization arm (for details, see main text). **a** Disease-free survival (DFS) at 10 years, **b** probability of overall survival (pOS) at 10 years, **c** cumulative incidence (CI) of relapses and deaths in CR at 10 years. **d** Disease-free survival (DFS) at 10 years only patients randomized to DEXA during induction, **e** disease-free survival (DFS) at 10 years only patients randomized to MePRED during induction. *ASP* asparaginase, *CI* cumulative incidence, *CR* complete remission, *CCR* continuous CR, *DFS* disease-free survival, *IM* intramuscular, *pOS* probability of overall survival

No difference was found between randomization arms with respect to total relapse rates (17.8% and 17.4%, respectively; *p* = 0.96), relapse sites, and incidences of secondary malignancies (Table 3). Also, probabilities of disease-free survival (DFS) at 10 years were not significantly different (arm ASP-5000: 79 ± 2%, arm ASP-10000: 75 ± 2%; *p*_log-rank_ = 0.155; Fig. 2a). The probability of overall survival (pOS) was slightly superior for patients of arm ASP-5000 compared to arm ASP-10000 (86 ± 2% and 82 ± 2%, respectively; *p*_log-rank_=0.07; Fig. 2b). The cumulative incidence (CI) of relapse was similar in both arms (Fig. 2c). An advantage of the higher ASP dose was not found in any of the groups. In contrast, DFS was significantly superior in boys, and OS higher in boys and young children (aged ≥ 1–< 5 years) in arm ASP-5000. DFS was independent of the glucocorticoid used during induction (DEXA vs. MePRED) (Table 4; Fig. 2d, e). In contrast, CI of treatment-related mortality (TRM) was significantly higher in boys and young children (aged ≥ 1–<5 years) as well as older children (aged ≥ 10 years) in arm ASP-10000 (Supplementary Table 1). Death in CR was mainly caused by infections (with comparable incidence in neutropenic and non-neutropenic patients) and occurred significantly less frequently in arm ASP-5000 (*n* = 9, 2.7%) than arm ASP-10000 (*n* = 23, 6.5%) (*p* = 0.029) (Table 5). Infections were diagnosed based on clinical findings. Most patients had been suffering from severe, rapidly progressing bacterial and/or fungal infections. Pneumonia was seen in about 50% of patients; two children in arm ASP-10000 died from fulminant reactivation of hepatitis B. Differences in timepoint of deaths are shown in Table 5. In arm ASP-10000, more than 60% of treatment-related deaths (TRD) were seen late (in consolidation III and maintenance therapy), whereas about 80% of TRD in arm ASP-5000 occurred early (during consolidation I and II). The incidences of pancreatitis and thrombosis were comparable between randomization arms.

**Table 3:** Treatment results by randomization arm

	Arm ASP-5000		Arm ASP-10000		*p*
	*n*	%	*n*	%	
Total patients	334	100	354	100	
All relapses	58	17.4	63	17.8	0.961
Site					
Bone marrow	37	11.1	33	9.3	0.525
CNS	5	1.5	10	2.8	0.352
Testis	1	0.3	4	1.1	0.405
BM + CNS	12	3.6	12	3.4	0.950
BM + testis	3	0.9	4	1.1	0.938
Other	0	0.0	0	0.0	–
Secondary malignancy	2	0.6	2	0.6	0.658
Death in CR	9	2.7	23	6.5	**0.029**
LFU	5	1.5	9	2.5	0.389
Continuous CR	**260**	**77.8**	**257**	**72.6**	**0.115**

*ASP* asparaginase, *BM* bone marrow, *CNS* central nervous system, *CR* complete remission, *IM* intramuscular, *LFU* lost to follow-up

**Table 4:** Characteristics and outcomes of SRG patients in the ALL-Moscow–Berlin 2002 trial by randomization arm

	Disease-free survival (DFS)		*р*	Overall survival (OS)		*р*
	Arm ASP-5000	Arm ASP-10000		Arm ASP-5000	Arm ASP-10000	
Gender						
Boys	80 ± 3%	72 ± 3%	**0.03**	84 ± 4%	81 ± 3%	**0.05**
Girls	78 ± 3%	80 ± 3%	0.81	84 ± 3%	83 ± 3%	0.83
Age (years)						
≥ 1–< 5	84 ± 3%	79 ± 3%	0.20	88 ± 4%	83 ± 3%	**0.04**
≥ 5–< 10	78 ± 4%	76 ± 4%	0.72	83 ± 4%	85 ± 4%	0.70
≥ 10	70 ± 5%	63 ± 6%	0.27	80 ± 5%	74 ± 5%	0.23
Initial white blood cell count						
< 10,000/µl	80 ± 3%	76 ± 3%	0.25	85 ± 3%	83 ± 3%	0.23
≥ 10,000–< 30,000/µl	78 ± 4%	77 ± 4%	0.82	81 ± 6%	84 ± 4%	0.78
≥ 30,000–< 50,000/µl	77 ± 9%	64 ± 8%	0.28	86 ± 7%	70 ± 8%	0.16
Genetics						
*t*(12;21)	93 ± 5%	88 ± 7%	0.51	83 ± 13%	100 ± 0%	0.24
Induction therapy						
Dexamethasone 6 mg/m^2^	80 ± 3%	78 ± 3%	0.49	84 ± 3%	83 ± 3%	0.42
Methylprednisolone 60 mg/m^2^	79 ± 3%	72 ± 3%	0.17	85 ± 4%	80 ± 3%	0.12

DFS was significantly superior in boys, and OS higher in boys and young children (aged ≥ 1–< 5 years) in the arm ASP-5000 than in the arm ASP-10000. DFS was independent of other patient characteristics shown, in particular of the glucocorticoid used during induction (Dexamethasone vs. Methylprednisolone)

Statistical significant *р* values are in bold

**Table 5:** Treatment-related death (TRD) of patients with standard-risk childhood acute lymphoblastic leukemia (ALL) in complete remission (CR) in the ALL-Moscow–Berlin 2002 trial by randomization arm

	Arm ASP-5000		Arm ASP-10000		*P*_Fisher_
	*n*	%	*n*	%	
TRD in CR	**9**	**100**	**23**	**100**	***0.029***
Time of death					
Day of therapy, median [interquartile range]	158 [116–163]		220 [100–264.75]		*0.213*
Consolidation I	4	44.4	8	34.8	*0.696*
Consolidation II	3	33.3	1	4.3	*0.057*
Consolidation III	1	11.1	8	34.8	*0.383*
Maintenance therapy	1	11.1	6	26.1	*0.640*

TRD in CR occurred significantly less frequently in arm ASP-5000 (9/334 patients) than arm ASP-10000 (23/354 patients). In arm ASP-10000, more than 60% of TRD were seen late (in consolidation III and maintenance therapy), whereas about 80% of TRD in arm ASP-5000 occurred early (during consolidation I and II)

Statistical significant *р* values are in bold

Severe HSR occurred significantly more frequently in arm ASP-10000 than ASP-5000 (4.5% vs. 1.8%; *p* = 0.07). In 7 patients of arm ASP-5000 and in more than twice as many (*n* = 16) patients who received 10,000 U/m^2^, ASP had to be discontinued. Among patients of arm ASP-5000, the reasons for discontinuation were: HSR (*n* = 4); pancreatitis (*n* = 2) and severe, persistent vomiting (*n* = 1), whereas HSR (*n* = 13) and pancreatitis (*n* = 3) led to discontinuation of ASP for patients in arm ASP-10000. Estimates of DFS “as treated” at 10 years were 80 ± 2% in arm ASP-5 and 74 ± 2% in arm ASP-10 (*p*(log-rank) = 0.11); OS probabilities were 86 ± 2% and 80 ± 2%, respectively (*p*_log-rank_ = 0.06) (data not shown).

## Discussion

In this randomized multicenter trial across Russia and Belarus, reduction of the ASP dose from 10,000 U/m^2^ to 5000 U/m^2^ IM did not have any negative effect on the outcome of more than 300 SR pediatric patients with ALL. Estimates of DFS were comparable and side effects, in particular TRM, were seen more frequently in patients who received the higher dose. The DFS of 79 ± 2% is similar to that reported by previous studies, in which SR patients comprised only about 30% of the total cohort compared to 70% in our study (Schrappe et al. 2000; Pession et al. 2005; Silverman et al. 2010). The weekly dose of 5000 U/m^2^ IM proved to be safe and cost-efficient. Therefore, only 5000 U/m^2^ are being used in subsequent trials in Russia.

Nevertheless, the results of this trial raise some important questions. ASP preparations have been used at a wide variety of dosages starting at 5000 U/m^2^, in early trials even at lower or higher doses up to 30,000 U/m^2^, at different time intervals and also via varying routes of administration (Pession et al. 2005; Muller and Boos 1998; Albertsen et al. 2001; Schrey et al. 2010). The mechanism of action associated with antileukemic ASP treatment is the ASP-provided hydrolyzation of asparagine (ASN) in the patient’s plasma. It is assumed that complete and sustained ASN depletion is required to achieve optimal ASP treatment effects. Monitoring ASP activity or asparagine levels, respectively, has been performed with conflicting results: According to most reports, trough levels of ASP should not fall below 100 U/L in order to achieve “complete” depletion but even lower levels, < 50 U/L, were suggested to be sufficient by others (Boos et al. 1996; Riccardi et al. 1981; Rizzari et al. 2000). Thus, the exactly required minimum serum enzyme activity remains unclear. In addition, there is still uncertainty about the extent of ASN depletion. On the one hand, the accuracy of monitoring ASN levels depends on the sensitivity of the method, which is limited; on the other hand, monitoring data may not be valid after all, because ASN depletion does continue in vitro after blood sampling, despite immediate cooling (Lanvers-Kaminsky et al. 2014). If “complete” depletion is a realistic option, it is not clear whether this should be achieved during induction or as part of post-induction therapy or both.

Usually, ASP is part of the induction therapy, but has been used for intensification during consolidation and maintenance therapy as well. About 10% improvement in EFS was reported by the administration of 20 additional weekly doses of (predominantly) *Erwinia*-ASP during maintenance therapy in SR patients (Pession et al. 2005). In contrast, no positive effect on outcome was seen after high-dose ASP in SR patients in a Dutch study (Kamps et al. 2002). Also, no advantage was shown after additional administration of *Erwinia*-ASP during maintenance in intermediate risk patients or *E. coli*-ASP during consolidation therapy in another study, respectively (Schrappe et al. 2000; Rizzari et al. 2001).

In addition to the question during which phase of therapy ASN depletion is relevant, it is not clear for what period of time it is required. Based on the currently available data, permanent trough levels of ≥ 100 IU/L can most likely be achieved with ASP at a dose of 5000 U/m^2^ IV three times weekly (Schrey et al. 2010).

Unfortunately, no prospective controlled study comparing the pharmacology after IV vs. IM *E. coli*-ASP has been published yet. Comparison of IV PEG-ASP with IM *E. coli*-ASP showed similar efficacy and toxicity, but the IV route was associated with decreased anxiety (Place et al. 2015). The recommended dose of native *E. coli*-ASP is 5000 U/m^2^ per injection site corresponding to a volume of 2 ml. It is not surprising that IM injections of higher doses are not appreciated by pediatric patients. Nevertheless, reliable data comparing the pharmacology of IM and IV administered ASP are lacking. In a recent report, about 20% of patients who received 25,000 U/m^2^ native *E. coli*-ASP IM weekly had nadir serum ASP activities lower than 100 U/L (Vrooman et al. 2013). Thus, permanent and complete depletion can hardly be expected in all patients. Likewise, sustained and complete ASN depletion cannot be expected after IM administration of 25,000 U/m^2^*Erwinia*-ASP because of its inferior activity compared with *E. coli-*ASP (Pession et al. 2005; Asselin et al. 1993; Duval et al. 2002). A general question remains whether “total” ASN depletion with the consequence of inhibiting protein synthesis might diminish the sensitivity of leukemic cells to other cytotoxic drugs.

The purpose of this study was not to investigate the pharmacokinetics of ASP. Hence, ASP activities were not routinely monitored during the course of our study. However, in the subsequent trial ALL-MB 2008, serum samples have been randomly collected and enzyme activities measured in Muenster (Prof. Boos). As shown in supplementary Fig. 2a and 2b, ASP trough levels (day 7) were far below 100 U/L in most patients. Thus, in the MB regimen, the supposedly required trough levels of ASP activity are neither achieved after 5000 U/m^2^ nor after 10,000 U/m^2^. Since the DFS and OS results in our study correspond to international published data in SR patients, we may conclude that sustained trough levels of ≥ 100 U/L ASP activity may not be necessary for the antileukemic efficacy of the drug. Alternatively, one could question whether in context with the MB protocol design, ASP is necessary at all—an assumption which is rather unlikely, however.

Nevertheless, despite the relatively low dose of ASP, the weekly dosing regimen and the large size of the SR group (70% of the total cohort), the DFS rate of 80% in this study is high when compared to other trials using much higher doses of ASP. One reason might be that we used only Asparaginase medac™, which has been shown to be the most potent *E. coli*-ASP preparation (Boos et al. 1996; Rizzari et al. 2000). Another explanation might be the exceedingly low rate of allergic reactions in our study. HSR have been significantly more frequent after IV than IM administration—even if PEG-ASP was used (Nesbit et al. 1979; Petersen et al. 2014; Henriksen et al. 2015; Abbott et al. 2015; van der Sluis et al. 2016). Frequently, HSR to ASP are accompanied by silent inactivation, and a lower rate of such events may have contributed to the result of our study (van der Sluis et al. 2016; Asselin and Rizzari 2015). In a study of the Dana-Farber Cancer Institute (DFCI), independent positive effects were seen after post-induction DEXA and individualized dosing of *E. coli*-ASP (Vrooman et al. 2013); in this context, the frequent use of DEXA in our protocol could also have had a positive effect on outcome.

Toxicity, in particular TRM, was more frequent in the ASP-10000 arm. Although ASP is assumed not to have substantial hematological toxicity, a myelosuppressive effect of prolonged ASP therapy in consolidation requiring dose reductions of other critical chemotherapy agents has been reported (Merryman et al. 2012). Also, myelosuppressive effects of ASP in part interfering with antimetabolite therapy have been described earlier (Johnston et al. 1974; Salzer et al. 2007; Hijiya and van der Sluis 2016). Hematological toxicity, though not in detail documented in our trial, may represent a contributing factor to the increased late, mostly infection-related, TRM in the ASP-10000 arm as described above. As far as toxicity is concerned, a synergistic effect between ASP and glucocorticoids, in particular DEXA, has been described (Liu et al. 2016). The authors concluded from their studies in mice that ASP can potentiate the osteonecrotic effects of glucocorticoids. Epiphyseal arteriopathy, an initiating event for osteonecrosis, was observed in 58% of mice receiving ASP and DEXA compared to 17% of mice receiving DEXA only. Previously, we have described a very low incidence of avascular necrosis of bone (AVN) despite high cumulative doses of DEXA in our protocol (Karachunskiy et al. 2015). In context with the observed association between ASP and avascular necrosis (AVN), this may be explained by the modest dose of 6 mg/m^2^ DEXA and the limited use of ASP.

Overall, our study confirms that numerous questions concerning ASP therapy are still open. Reports of clinical trials and also overviews should always clearly indicate the source of the drugs used, as well as the dose, route of administration, dosing intervals, and details of the chemotherapy protocol to allow valid comparisons and interpretations. According to a recent consensus, the most reliable parameter to assess clinical effects is monitoring of ASP serum activity after defined time intervals (van der Sluis et al. 2016). If necessary, therapy could then be adapted to individual metabolism and tolerance.

The regimen used in this study proved to be effective in SR patients comprising 70% of the total group and defined by the very simple stratification system. If we applied the refined stratification of the subsequent trial ALL-MB 2008, the SR group would be reduced to 50% of the total group, and for these patients, DFS would yield 86 ± 3% for the ASP-5000 arm (Suppl. Fig. 3A). Comparably favorable DFS rates can be calculated when stratifying our SRG patients according to SR criteria used by the International Berlin-Frankfurt-Muenster-(IBFM) group (Suppl. Fig. 3B) or by the DFCI (Suppl. Fig. 3C), respectively. Of note, the MB protocol contained very moderate anthracycline doses and no high-dose or substantially cytotoxic therapies, e.g., HD methotrexate or cyclophosphamide.

In summary, the ASP dose of 5000 U/m^2^ was not associated with inferior results compared to 10,000 U/m^2^. HSR rates and adverse effects were overall low in both arms. IM-injection volumes were moderate and well tolerated. Favorable results were achieved with the lower ASP dose at defined dosing intervals. Critical evaluation of the results suggests that some assumptions concerning duration and completeness of ASN depletion and correspondingly required enzyme levels do not match current opinions and principles. Obviously, it is possible to reduce the ASP dose if the overall antileukemic power of the protocol is high. With respect to cure rates as well as economical and ethical considerations, such as preventing toxicity and being aware of cost-efficiency, the ASP-5000 arm in context with the MB treatment design is a reasonable therapeutic approach for pediatric patients with SR-ALL—not only in countries with limited resources.

## Electronic supplementary material

Below is the link to the electronic supplementary material.

***Supplementary Figure 1***. Overall Study Design of ALL-MB 2002 (TIF 149 KB)

***Supplementary Figure 2a and 2b***. Asparaginase (ASP) levels day 3 (2a) and day 7 (2b) after 5000 U/m^2^ and 10000 U/m^2^ Asparaginase medac^TM^ IM in serum of samples collected from patients treated in trial MB 2008 (Trial-Registry No. NCT01953770). Included were patients who had at least 2 measurements on day 3 and day 7 each, i.e. at least 4 measurements. Day 3 (N = 100 after 5000 U/m^2^, 897 samples; N = 113 after 10000 U/m^2^, 968 samples); day 7 (N = 100 after 5000 U/m^2^, 837 samples; N = 113 after 10000 U/m^2^, 915 samples). On day 3, ASP levels of ≥ 100 U/L were measured in 81% and 88% of samples after 5000 U/m^2^ and 10000 U/m^2^, respectively, (p < 0,01). On day 7, ASP levels of ≥ 100 U/L were found in 10% and 16% of samples after 5000 U/m^2^ and 10000 U/m^2^, respectively, (p < 0,01). (PDF 79 KB)

***Supplementary Figure 3A***. Probability of disease-free survival (pDFS) for standard risk group (SRG, as defined below in protocol ALL-MB 2008) patients in trial ALL-MB 2002 stratified according to criteria of the “revised” version of protocol ALL-MB 2008 (SRG = 50% of total cohort). *SRG criteria (as per protocol ALL-MB 2008)*: patients - WBC<30,000/mm^3^, no CNS involvement, spleen<4cm below costal margin, non-T-cell ALL, no t(4;11) and t(9;22), remission on day 36. *Source*: ALL-MB 2008 treatment protocol, Trial-Registry No.: NCT01953770. *Abbreviations*: ALL – acute lymphoblastic leukemia, ASP – asparaginase, CNS – central nervous system, MB – Moscow Berlin, WBC – white blood cell count. (JPG 50 KB)

***Supplementary Figure 3B***. Probability of DFS (pDFS) for standard risk group (SRG, as defined below by the Berlin-Frankfurt-Muenster (BFM) group) patients in trial ALL-MB 2002 stratified according to criteria of the International (I) -BFM group. *SRG criteria (as per BFM group)*: patients - 1-15 years old at diagnosis, primary non-T-cell ALL, BFM risk factor < 0.8, good response on day 8, (<1000 leukemic blasts/mm^3^), no t(4;11) and t(9;22), remission on day; treatment *- Erwinia*-ASP – 20 weekly doses of 25,000 U/m^2^; results – pDFS with *Erwinia*-ASP (at 10 years) is 87.5±2.5%. *Source*: Pession A., Valsecchi MG, Masera G, Kamps WA, et al. Long-Term Results of a Randomized Trial on Extended Use of High Dose L-Asparaginase for Standard Risk Childhood Acute Lymphoblastic Leukemia. *J Clin Oncol* 2005, 23(28):7161-7167. *Abbreviations*: ALL – acute lymphoblastic leukemia, ASP – asparaginase, EFS – event-free survival, MB – Moscow-Berlin. (JPG 51 KB)

***Supplementary Figure 3C***. Probability of disease-free survival (pDFS) for standard risk group (SRG, as defined below by the Dana Farber Cancer Institute (DFCI)) patients in trial ALL-MB 2002 stratified according to criteria of the DFCI. *SRG criteria (as per DFCI, protocol 91-01)*: patients - 2-9 years old, primary non-T-cell ALL, WBC<20,000/mm^3^, no CNS involvement, no t(9;22); treatment – *E. coli*-ASP 30 weekly 25,000 U/m^2^; results – EFS with *E. coli*-ASP (at 12 years) is 83.2±3.3%. *Source*: Silverman LB., Stevenson KE., O’Brien JE. et al. Long-term results of Dana-Farber Cancer Institute ALL Consortium protocols for children with newly diagnosed acute lymphoblastic leukemia (1985–2000). *Leukemia* 2010; 24(2): 320-334). *Abbreviations*: ALL – acute lymphoblastic leukemia, ASP – asparaginase, CNS – central nervous system, EFS – event-free survival, MB – Moscow-Berlin, WBC – white blood cell count. (JPG 49 KB)

Supplementary material 6 (DOCX 18 KB)
