# Supplementary figures and images for: Reduced vs. standard dose native E. coli-asparaginase therapy in childhood acute lymphoblastic leukemia: long-term results of the randomized trial Moscow–Berlin 2002
Source: J Cancer Res Clin Oncol. 2019 Mar 6;145(4):1001–12. doi: 10.1007/s00432-019-02854-x (PMC6435612; doi:10.1007/s00432-019-02854-x)

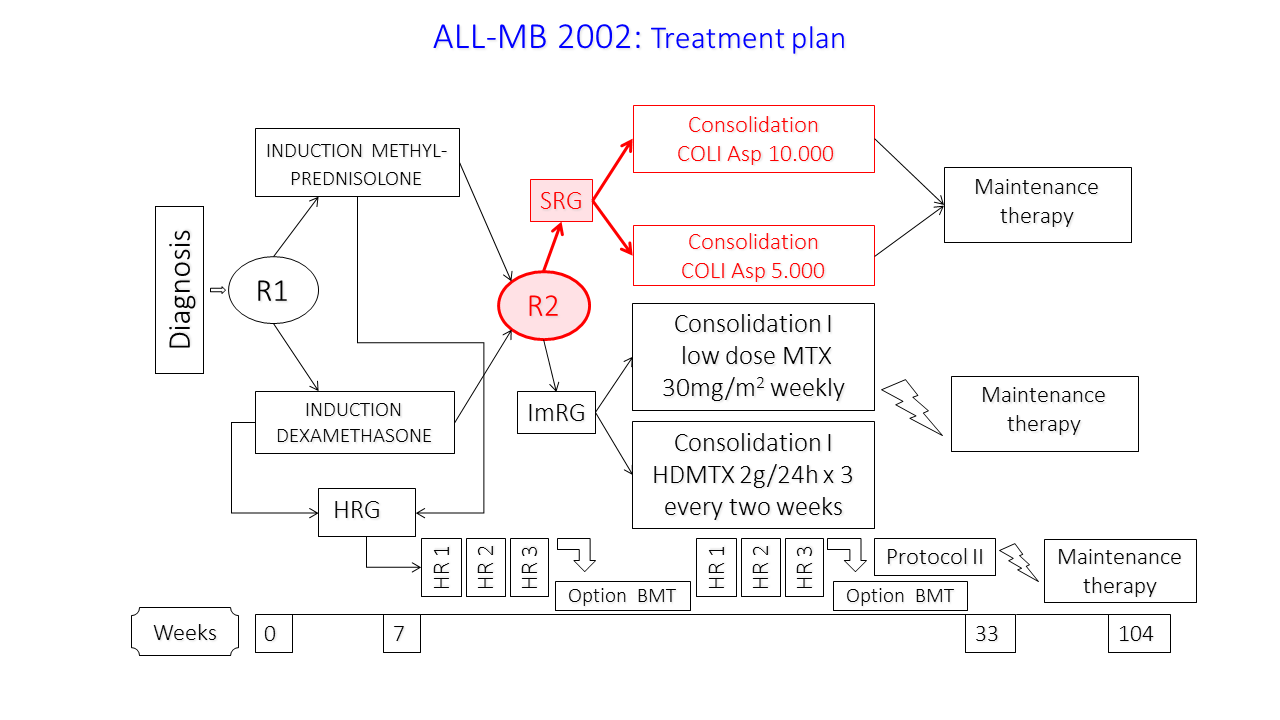

Supplement: Supplementary file 1 — Supplementary Figure 1. Overall Study Design of ALL-MB 2002 (TIF 149 KB) [file 432_2019_2854_MOESM1_ESM.tif]

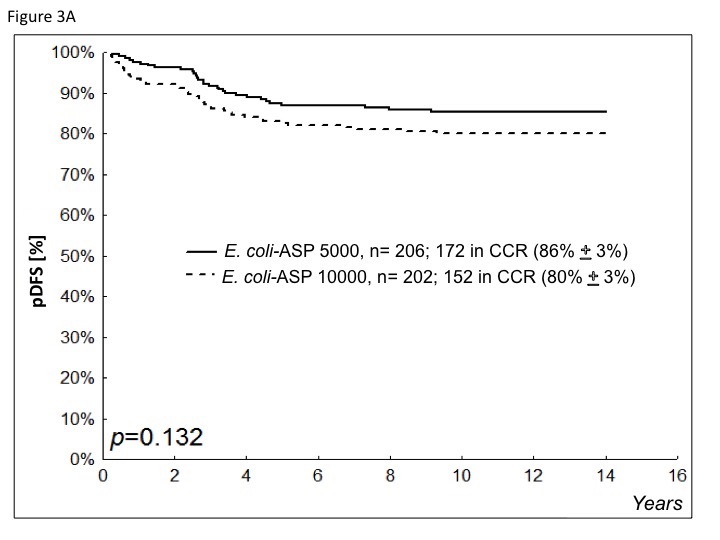

Supplement: Supplementary file 3 — Supplementary Figure 3A. Probability of disease-free survival (pDFS) for standard risk group (SRG, as defined below in protocol ALL-MB 2008) patients in trial ALL-MB 2002 stratified according to criteria of the “revised” version of protocol ALL-MB 2008 (SRG = 50% of total cohort). SRG criteria (as per protocol ALL-MB 2008): patients - WBC<30,000/mm3, no CNS involvement, spleen<4cm below costal margin, non-T-cell ALL, no t(4;11) and t(9;22), remission on day 36. Source: ALL-MB 2008 treatment protocol, Trial-Registry No.: NCT01953770. Abbreviations: ALL – acute lymphoblastic leukemia, ASP – asparaginase, CNS – central nervous system, MB – Moscow Berlin, WBC – white blood cell count. (JPG 50 KB) [file 432_2019_2854_MOESM3_ESM.jpg]

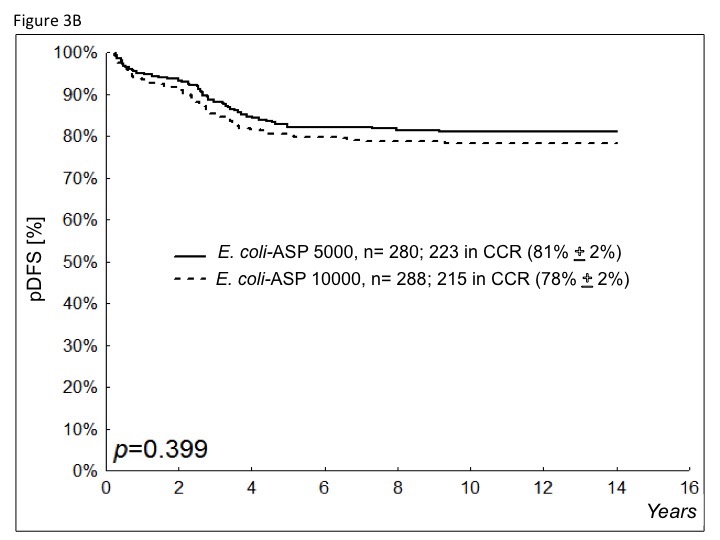

Supplement: Supplementary file 4 — Supplementary Figure 3B. Probability of DFS (pDFS) for standard risk group (SRG, as defined below by the Berlin-Frankfurt-Muenster (BFM) group) patients in trial ALL-MB 2002 stratified according to criteria of the International (I) -BFM group. SRG criteria (as per BFM group): patients - 1-15 years old at diagnosis, primary non-T-cell ALL, BFM risk factor < 0.8, good response on day 8, (<1000 leukemic blasts/mm3), no t(4;11) and t(9;22), remission on day; treatment - Erwinia-ASP – 20 weekly doses of 25,000 U/m2; results – pDFS with Erwinia-ASP (at 10 years) is 87.5±2.5%. Source: Pession A., Valsecchi MG, Masera G, Kamps WA, et al. Long-Term Results of a Randomized Trial on Extended Use of High Dose L-Asparaginase for Standard Risk Childhood Acute Lymphoblastic Leukemia. J Clin Oncol 2005, 23(28):7161-7167. Abbreviations: ALL – acute lymphoblastic leukemia, ASP – asparaginase, EFS – event-free survival, MB – Moscow-Berlin. (JPG 51 KB) [file 432_2019_2854_MOESM4_ESM.jpg]

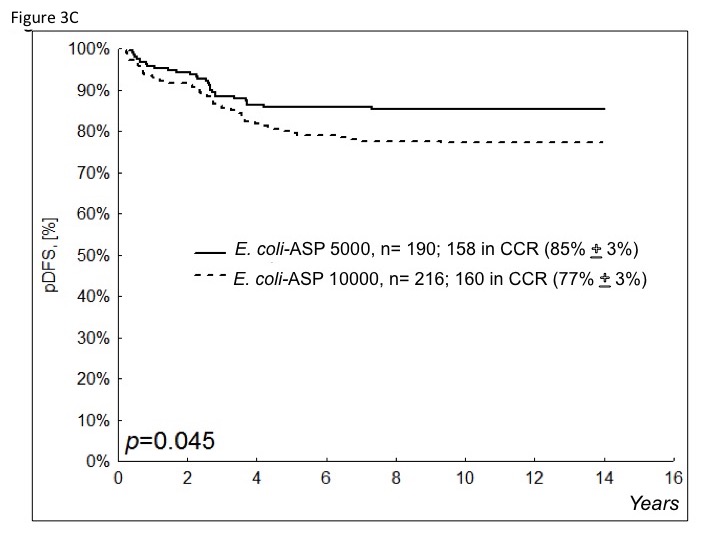

Supplement: Supplementary file 5 — Supplementary Figure 3C. Probability of disease-free survival (pDFS) for standard risk group (SRG, as defined below by the Dana Farber Cancer Institute (DFCI)) patients in trial ALL-MB 2002 stratified according to criteria of the DFCI. SRG criteria (as per DFCI, protocol 91-01): patients - 2-9 years old, primary non-T-cell ALL, WBC<20,000/mm3, no CNS involvement, no t(9;22); treatment – E. coli-ASP 30 weekly 25,000 U/m2; results – EFS with E. coli-ASP (at 12 years) is 83.2±3.3%. Source: Silverman LB., Stevenson KE., O’Brien JE. et al. Long-term results of Dana-Farber Cancer Institute ALL Consortium protocols for children with newly diagnosed acute lymphoblastic leukemia (1985–2000). Leukemia 2010; 24(2): 320-334). Abbreviations: ALL – acute lymphoblastic leukemia, ASP – asparaginase, CNS – central nervous system, EFS – event-free survival, MB – Moscow-Berlin, WBC – white blood cell count. (JPG 49 KB) [file 432_2019_2854_MOESM5_ESM.jpg]
