# Supplementary material for: Reduced vs. standard dose native E. coli-asparaginase therapy in childhood acute lymphoblastic leukemia: long-term results of the randomized trial Moscow–Berlin 2002
Source: J Cancer Res Clin Oncol. 2019 Mar 6;145(4):1001–12. doi: 10.1007/s00432-019-02854-x (PMC6435612; doi:10.1007/s00432-019-02854-x)
Supplement: Supplementary file 2 — Supplementary Figure 2a and 2b. Asparaginase (ASP) levels day 3 (2a) and day 7 (2b) after 5000 U/m2 and 10000 U/m2 Asparaginase medacTM IM in serum of samples collected from patients treated in trial MB 2008 (Trial-Registry No. NCT01953770). Included were patients who had at least 2 measurements on day 3 and day 7 each, i.e. at least 4 measurements. Day 3 (N = 100 after 5000 U/m2, 897 samples; N = 113 after 10000 U/m2, 968 samples); day 7 (N = 100 after 5000 U/m2, 837 samples; N = 113 after 10000 U/m2, 915 samples). On day 3, ASP levels of ≥ 100 U/L were measured in 81% and 88% of samples after 5000 U/m2 and 10000 U/m2, respectively, (p < 0,01). On day 7, ASP levels of ≥ 100 U/L were found in 10% and 16% of samples after 5000 U/m2 and 10000 U/m2, respectively, (p < 0,01). (PDF 79 KB) [file 432_2019_2854_MOESM2_ESM.pdf]

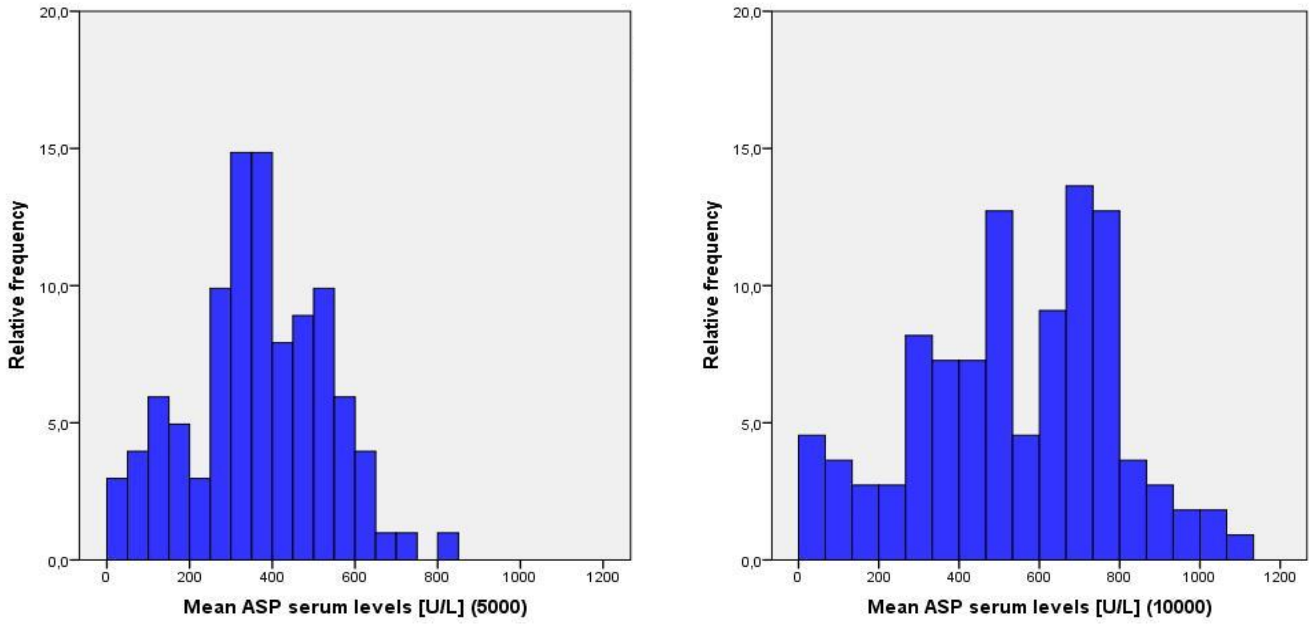

2a

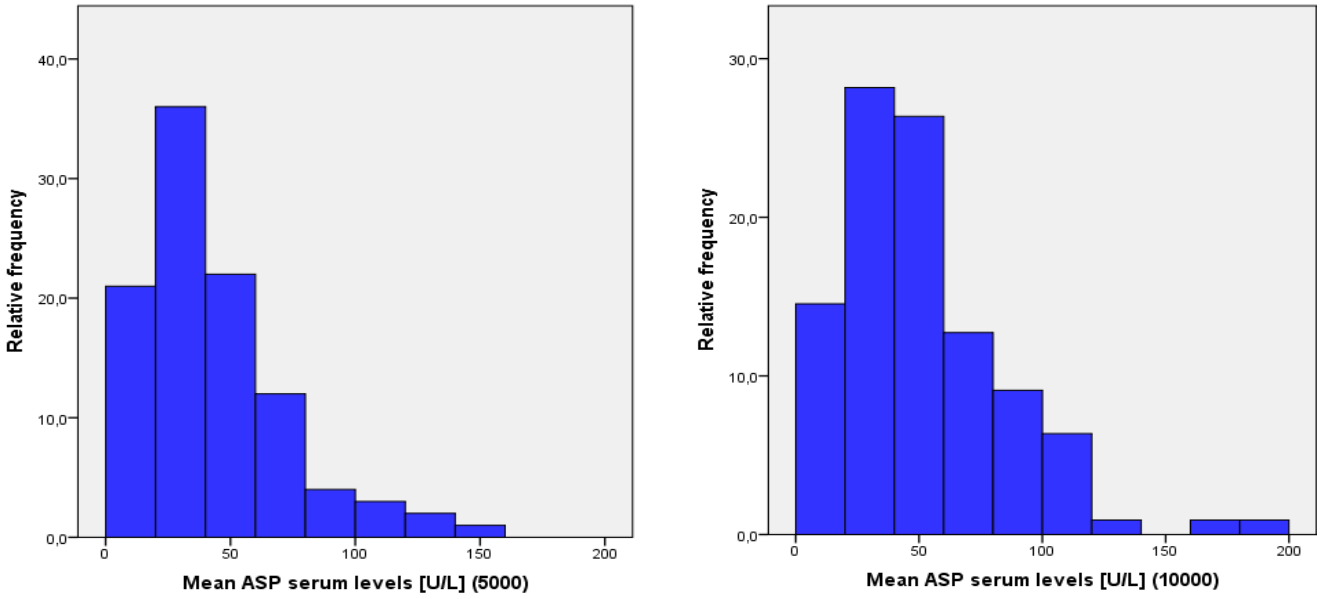

2b

Supplementary Fig. 2 a and b

Asparaginase (ASP) levels day 3 (2a) and day 7 (2b) after 5000 U/m^2 and 10000 U/m^2 Asparaginase medac^{TM} IM in serum of samples collected from patients treated in trial MB 2008 (Trial-Registry No. NCT01953770). Included were patients who had at least 2 measurements on day 3 and day 7 each, i.e. at least 4 measurements. Day 3 (N = 100 after 5000 U/m^2, 897 samples; N = 113 after 10000 U/m^2, 968 samples); day 7 (N = 100 after 5000 U/m^2, 837 samples; N = 113 after 10000 U/m^2, 915 samples).

On day 3, ASP levels of ≥ 100 U/L were measured in 81% and 88% of samples after 5000 U/m^2 and 10000 U/m^2, respectively, ($p < 0,01$). On day 7, ASP levels of ≥ 100 U/L were found in 10% and 16% of samples after 5000 U/m^2 and 10000 U/m^2, respectively, ($p < 0,01$).
