# Supplementary material for: Reduced vs. standard dose native E. coli-asparaginase therapy in childhood acute lymphoblastic leukemia: long-term results of the randomized trial Moscow–Berlin 2002
Source: J Cancer Res Clin Oncol. 2019 Mar 6;145(4):1001–12. doi: 10.1007/s00432-019-02854-x (PMC6435612; doi:10.1007/s00432-019-02854-x)
Supplement: Supplementary file 6 — Supplementary material 6 (DOCX 18 KB) [file 432_2019_2854_MOESM6_ESM.docx]

**Supplementary table 1.** Patient characteristics and cumulative incidences (CI) of relapse and treatment-related mortality (TRM) in children with standard-risk childhood acute lymphoblastic leukemia (ALL) in the ALL-Moscow-Berlin 2002 trial by randomization arm.

|  | **CI of relapse** | | ***р*** | **CI of TRM** | | ***р*** |
| --- | --- | --- | --- | --- | --- | --- |
|  | **Arm ASP-5000** | **Arm ASP-10000** |  | **Arm ASP-5000** | **Arm ASP-10000** |  |
| **Gender** | | | | | | |
| Boys | 17.9±2.9 | 20.1±2.9 | *0.5609* | 1.1±0.8 | 7.3±1.9 | ***0.0033*** |
| Girls | 16.4±3.0 | 14.9±2.8 | *0.7170* | 4.6±1.7 | 5.6±1.8 | *0.6957* |
| **Age** | | | | | | |
| ≥1 − <5 years | 12.4±2.6 | 12.3±2.4 | *0.9617* | 2.5±1.2 | 7.4±1.9 | ***0.0394*** |
| ≥5 − <10 years | 16.2±3.8 | 20.7±4.3 | *0.4274* | 5.4±2.4 | 3.3±1.9 | *0.4745* |
| ≥10 years | 28.3±5.2 | 28.4±5.5 | *0.9401* | 0 | 8.2±3.2 | ***0.0091*** |
| **Initial white blood cell count** | | | | | | |
| <10,000/μl | 16.0±2.6 | 16.0±2.5 | *0.9511* | 2.9±1.2 | 7.1±1.7 | ***0.0470*** |
| ≥10,000 − <30,000/μl | 18.7±3.9 | 17.7±4.1 | *0.8180* | 2.9±1.7 | 5.3±2.3 | *0.4050* |
| ≥30,000 − <50,000/μl | 22.7±9.2 | 30.3±8.2 | *0.5598* | 0 | 6.1±4.2 | *0.2440* |
| **Spleen enlargement below costal margin** | | | | | | |
| <4 cm | 13.4±2.1 | 16.8±2.4 | *0.2771* | 3.1±1.1 | 6.3±1.5 | ***0.0941*** |
| ≥4 cm | 30.3±5.4 | 20.4±4.1 | *0.1440* | 1.3±1.3 | 7.1±2.6 | ***0.0699*** |
| **Genetics** | | | | | | |
| t(12;21) | 6.9±4.6 | 8.3±5.8 | *0.8450* | 0 | 0 | *na* |
| **Response to treatment on day 8** | | | | | | |
| <1,000 leukemic blasts/μl PB | 17.5±2.1 | 15.5±2.0 | *0.5269* | 2.2±0.8 | 6.7±1.4 | ***0.0064*** |
| ≥1,000 leukemic blasts/μl PB | 11.1±11.1 | 66.1±14.4 | ***0.0166*** | 11.1±11.1 | 7.1±7.1 | *0.7227* |
| **Response to treatment on day 15** | | | | | | |
| <10% leukemic blasts in BM | 14.5±2.2 | 14.9±2.2 | *0.7943* | 2.2±0.9 | 6.7±1.5 | ***0.0114*** |
| ≥10 − <25% leukemic blasts BM | 27.5±7.2 | 25.6±7.1 | *0.8452* | 7.5±4.2 | 2.6±2.6 | *0.3172* |
| ≥25% leukemic blasts in BM | 31.6±11.0 | 29.2±9.5 | *0.9990* | 0 | 8.3±5.8 | *0.2032* |
| **Induction therapy** | | | | | | |
| Dexamethasone 6 mg/m^2^ | 18.3±3.0 | 15.4±2.7 | *0.5512* | 0.6±0.6 | 6.3±1.8 | ***0.0043*** |
| Methylprednisolone 60 mg/m^2^ | 16.6±2.9 | 20.3±3.0 | *0.4159* | 4.3±1.6 | 6.8±1.9 | *0.3304* |

***Abbreviations:*** BM – bone marrow, PB – peripheral blood
